# Association between dietary fiber intake and bone mineral density: a systematic review and meta-analysis of observational studies

**DOI:** 10.1007/s00394-025-03866-9

**Published:** 2026-02-25

**Authors:** Yuqi Pang, Zilan Chen, Ye Ju, Bin Yang, Jiaojiao Hou, Sirui Zheng, Zihao Li, Ting Liu, Hongxia Xia, Maoyao Xia, Yangdan Zhong, Jiayuan Li, Zhong Li, Xia Jiang

**Affiliations:** 1 Department of Nutrition and Food Hygiene and West China-PUMC C. C. Chen Institute of Health, West China School of Public Health and West China Fourth Hospital, Sichuan University, No. 16, Section 3, South Renmin Road, Wuhou District, Sichuan 610041 Chengdu, China; 2 Department of Epidemiology and Biostatistics, Institute of Systems Epidemiology, West China School of Public Health, West China Fourth Hospital, Sichuan University, No.16, Section 3, South Renmin Road, Wuhou District, Sichuan 610041 Chengdu, China; 3 Department of Toxicosis/Nephrology, West China School of Public Health, West China Fourth Hospital, Sichuan University, No.16, Section 3, South Renmin Road, Wuhou District, Sichuan 610041 Chengdu, China; 4 Department of Clinical Neuroscience, Karolinska Institute, Stockholm, Sweden

**Keywords:** Dietary fiber, Diet, Bone mineral density, Meta-analysis

## Abstract

**Background:**

Although prior studies have linked dietary fiber to bone mineral density (BMD), the currently available evidences remain inconsistent and there is a lack of synthesis. This systematic review and meta-analysis aimed to comprehensively examine the association between dietary fiber intake and BMD in adults.

**Methods:**

We systematically searched the PubMed, Ovid, Web of Science, and ScienceDirect for studies evaluating the association between dietary fiber and BMD, from 2000 to January 2025. Two authors independently extracted data and assessed the risk of bias using the NOS and the AHRQ for observational cohort and cross-sectional Studies. Pooled $\:\beta\:$-coefficients values and their corresponding 95%CIs were calculated using a random-effects model. Subgroup analyses were performed to explore potential sources of heterogeneity.

**Results:**

After a systematic search, 6 articles were included involving 7 studies. Meta-analyses included 4 cross-sectional studies with 229,339 individuals, while 2 cohort studies involving 3174 individuals and 1 cross-sectional study involving 9871 individuals were reviewed qualitatively. Overall results indicated that individuals with higher dietary fiber intake exhibited significantly higher BMD levels ($\:\beta\:$ = 0.013, 95%CI = 0.011–0.015, $\:P$ < 0.01; $\:{\tau\:}^{2}$ = 0.00; $\:{I}^{2}$ = 0.00%; $\:{P}_{Q-test}$ = 0.86). Leave-one-out sensitivity analyses confirmed the robustness of findings. Subgroup analyses revealed region as the source of heterogeneity. No evidence of publication bias was detected.

**Conclusion:**

This study indicates that higher dietary fiber consumption is significantly associated with higher BMD, particularly among males and populations outside Europe and America. Large-scale prospective cohort studies are needed to validate our findings.

**Supplementary Information:**

The online version contains supplementary material available at 10.1007/s00394-025-03866-9.

## Introduction

Osteoporosis is a prevalent chronic disease characterized by systemic deterioration in bone mass and microstructures, leading to an increased risk of fractures [1, 2]. Worldwide, osteoporotic fractures affect approximately 33% of women and 20% of men aged over 50 [3]. As global population ages, the impairment of physical function, comorbidity, and mortality associated with osteoporosis is expected to rise, thereby bringing a huge medical and social burden [2, 4]. To address this pressing public health challenge, it is important to identify modifiable factors associated with osteoporosis.

Bone mineral density (BMD) is the primary diagnostic indicator of osteoporosis and is influenced by a multitude of genetic and environmental factors [5]. Dietary modification is one of the few safe and easily implementable strategies. Maintaining a healthy and balanced nutritional intake has been shown to play a vital role in preventing the onset of osteoporosis as well as in slowing its progression [6]. Several prospective cohort studies have demonstrated that, in addition to vitamin D and calcium, dietary patterns rich in fruits and vegetables are associated with higher BMD [7–9]. Given that dietary fiber is a fundamental component underlying these foods, it is plausible to hypothesize a potential role of dietary fiber intake in the promotion of BMD. However, research on this topic remains limited, and the precise nature of the association remains unclear.

Several clinical trials have demonstrated that dietary fiber promotes calcium absorption [10, 11] and retention [12], but these benefits fail to be translated into measurable improvements in BMD for trials of middle-aged and elderly individuals ($\:{N}_{sample}$ 237–300, duration = 2 years) [13, 14]. Notably, in existing clinical trials, the term “dietary fiber” typically refers to a single type of fiber – such as wheat bran fiber or short-chain fructo-oligosaccharides – supplemented as an intervention. This neglects the fact that multiple types of fiber from daily diets may exert different biological effects compared to the single type. Furthermore, the duration of existing clinical trials does not exceed two years, which may be relatively short for dietary fiber interventions. Given that dietary fiber exerts its effects on bone health indirectly [15, 16] and that these effects require time to accumulate, significant improvements in BMD may only be observed over a longer duration [17, 18]. Therefore, evidence synthesis from well-designed, large-scale, population-based epidemiological studies is needed. However, findings to date remain inconsistent: smaller cross-sectional studies ($\:{N}_{sample}$ 300–500) reported null or negative associations [19, 20], whereas larger cross-sectional studies ($\:{N}_{sample}$ 2,800 − 220,000) suggested positive associations [21–23]. Longitudinal evidence also remains inconsistent: a Mexican cohort with 6-year follow-up ($\:{N}_{sample}$ = 1,317) observed a negative association between dietary fiber intake and femoral-neck BMD change in males [24], whereas the Framingham Offspring Study with 12-year follow-up ($\:{N}_{sample}$ = 1,857) found higher dietary fiber intake to be associated with more favorable annual changes in femoral-neck BMD among males [25].

Given the inconsistencies and limitations of existing research, as well as a lack of comprehensive synthesis, our study systematically reviews available data on the association between dietary fiber intake and BMD in adults and conducts a meta-analysis.

## Method

### Protocol and registration

This systematic review and meta-analysis was performed in accordance with the Preferred Reporting Items for Systematic Reviews and Meta-Analysis (PRISMA). A pre-specified protocol has been registered in the International Prospective Register of Systematic Reviews (PROSPERO) under the identification number CRD42025643323.

### Search strategy

A comprehensive literature search was conducted in PubMed, Ovid, Web of Science, and ScienceDirect databases from 2000 to January 2025 by two independent reviewers. The search was not restricted to any specific language. We used search terms including “Diet”, “Dietary fiber”, “bone mineral density”, “BMD”, “bone mineral content” and more. A detailed search strategy is provided in Supplementary Table S1. The reference lists of related reviews and articles were manually screened for additional eligible studies. All literature was imported into EndNote 21 for subsequent screening.

### Eligibility and study selection

Inclusion criteria were as follows: (1) the study was conducted in humans; (2) the study investigated the association between dietary fiber and bone mass – including bone mineral density, bone mineral content, T-score and Z-score; (3) the study reported effect estimates in a format of odds ratios (ORs), relative risks (RRs), hazard ratios (HRs), or $\:\beta\:$-coefficients, with corresponding 95% confidence intervals (95%CIs), and appropriate adjustments for confounders.

Exclusion criteria were as follows: (1) studies published in languages other than English; (2) studies targeting populations with specific comorbidities (e.g., cardiovascular disease, diabetes, or cancer) rather than the general population; (3) inappropriate types of publication (e.g., conference abstract or conference proceedings); (4) studies involving children or adolescents (i.e., aged 0–18 years); (5) studies with unavailable data. When multiple studies derived from the same cohort were identified, priority was given to the one with the greatest statistical power, i.e., the largest sample size, the most comprehensive adjustment for confounders, or the longest follow-up period.

Two authors (YP and ZC) independently assessed the eligibility of each study based on the pre-determined inclusion and exclusion criteria. After removing duplicates, titles and abstracts were screened to exclude irrelevant reports. Subsequently, the full texts of the remaining studies were evaluated in detail, with any discrepancies resolved through consultation with a third author (YJ) until consensus was achieved.

### Data extraction

Two reviewers (YP and YJ) independently extracted data from each eligible study, including the first author’s name, year of publication, study design, country, sample size, participants’ age and sex, methods used to assess dietary fiber intake and bone mass, covariates included in the statistical model, and the effect estimates with their 95%CIs. When multiple models with different adjustments were available, the most adjusted effect estimate was selected. All extracted data were cross-verified for consistency, and any discrepancies were resolved through discussion among the authors.

### Risk of bias assessment

Two reviewers (YP and ZC) independently evaluated the risk of bias for the included studies using the Newcastle-Ottawa Scale (NOS) [26] for cohort studies and the Agency for Healthcare Research and Quality tool (AHRQ) [27] for cross-sectional studies. Any discrepancies were resolved through discussion.

The NOS comprises eight items categorized into three dimensions: participant selection, comparability, and outcome determination. Each item within the selection and outcome category receives a maximum of one point, while comparability is worth a maximum of two points. Based on the calculated total scores, each study was classified as low (0–3 points), medium (4–6 points), or high (7–9 points) quality [28].

The AHRQ tool comprises 11 items, each categorized as “Yes”, “No”, or “Unclear”, with a score of 1 assigned for “Yes” and 0 for others. Total scores are then categorized as low (0–3 points), medium (4–7 points), or high-quality (8–11 points), in accordance with the most commonly used classification methods [29].

### Statistical analysis

According to WHO’s recommendation on that adults should obtain at least 25 g dietary fiber per day from food [30], the original $\:\beta\:$-coefficient corresponding to a 1 g/day dietary fiber as the unit of change was converted into an effect corresponding to a 25 g/day of dietary fiber as the unit of change. We used the pooled converted $\:\beta\:$-coefficients values and their corresponding 95%CIs to synthesize the results of each study by inverse variance weighting through a random effects model. Sensitivity analyses were performed using the leave-one-out elimination method to assess the robustness of results. Given the significant impact of calcium and vitamin D on bone health [31], additional sensitivity analyses were conducted by excluding studies that did not adjust for these key confounders.

We comprehensively evaluated the heterogeneity among studies (between-study heterogeneity) through three methods. First, the $I^{2}$ statistic was used to quantify the proportion of total variation attributable to heterogeneity, and an $\:{I}^{2}$ < 25%, 25–50% and > 50% was considered as low, moderate and high heterogeneity, respectively [32]. Second, the restricted maximum-likelihood estimator of $\:{\tau\:}^{2}$ was used to assess the heterogeneity [33]. Third, Q-tests were used to obtain the corresponding $\:P$-values. For meta-analyses involving fewer than 5 studies, we adopted the Hartung-Knap-Sidik-Jonkman (HKSJ) method to reduce the type I error rates [34].

Existing evidence suggests that the effects of nutrients on BMD are influenced by multiple key characteristics, such as ethnicity, levels of socio-economic development, and dietary patterns [9, 35–37], and may vary depending on BMD measurement site and dietary assessment methods [24, 38, 39]. Therefore, subgroup analyses were performed based on (1) BMD site (femoral neck vs. lumbar spine vs. hip), (2) sex (female vs. male), (3) regions (Europe and America vs. other), and (4) dietary assessment method (Food Frequency Questionnaire [FFQ] vs. 24 h-recall) to explore factors that might influence the association between dietary fiber intake and BMD. Publication bias was assessed using funnel plots and Begg’s tests. Statistical analyses and forest plots were conducted using software *R version 4.3.2.* A $\:P$-value < 0.05 was considered statistically significant.

## Results

### Literature search and selection

**Fig. 1:**
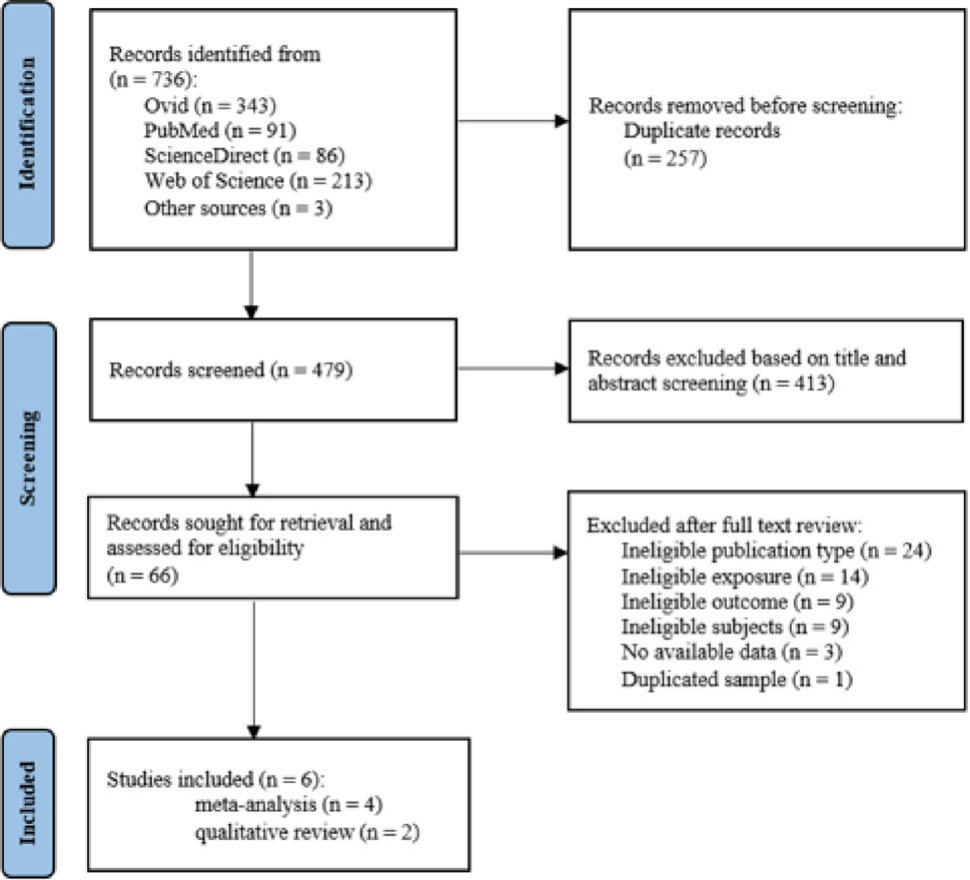
The overall flowchart of identification, screening and inclusion of studies

The flowchart of study selection is presented in Fig. 1. Initially, our comprehensive search retrieved a total of 736 records; after duplicate removal, 479 records remained for title and abstract screening. From these records, 66 reports were further reviewed for full-texts and assessed for eligibility. Ultimately, 6 original studies were included in our systematic review and meta-analysis. Detailed justifications for each excluded full-text report are provided in Supplementary Table S2.

### Study characteristics

Characteristics extracted from the included studies are summarized in Table 1. Notably, Rivera-Paredez’s study comprised two parts with distinct outcomes – a cross-sectional part reporting BMD and a longitudinal part reporting BMD change. Therefore, these two parts were treated as two separate studies. We ultimately included six articles involving seven studies, of which four studies reported BMD, one BMD change, 1% BMD change, and one T-score. Four studies reporting BMD as outcomes were included into meta-analysis, while the other three studies with slightly different outcomes were reviewed qualitatively.

**Table 1:** Characteristics of included studies

First author	Year	Country	Subject sex	Sample size	Male (%)	Age (mean ± SD or m, Q1-Q3 or range), y	Nutrient		Outcome			Covariates	Quality score
							Daily intake (mean ± SD or m, Q1-Q3),g/d	Measurement	Type	Site	Measurement		
Lushuang Zhang^2^	2024	USA	Female	2829	0.0	61.14 ± 0.23	15.61 ± 0.31	24-hr dietary recall data	BMD^1^	Femoral neck,	DXA^1^	Age, race, education, marriage, poverty-to-income ratio, physical activity, hypertension, previous fracture, BMI^1^, circumference, cotinine, protein intake, history of physician-diagnosed osteoporosis or osteoporosis treatment, estrogens treatment, and total energy	9
										Total femur			
B.Rivera-Paredez(A^2,3^	2023	Mexico	Female	990	0.0	46,37–55	25.9, (18.8–35.3)	FFQ^1^	BMD,	Femoral neck,	DXA	Age (years), BMI; energy intake, diabetes, smoking status, leisure-time physical activity, calcium intake, calcium supplements, vitamin D intake, UA levels^1^, alcohol intake, HRT^1^, caffeine intake	9
									BMD change	Lumbar spine,			
										Hip			
B.Rivera-Paredez(B^2,3^	2023	Mexico	Male	327	100.0	45,36–54	24.8, (18.1–34.1)	FFQ	BMD,	Femoral neck,	DXA	Age (years), BMI; energy intake, diabetes, smoking status, leisure-time physical activity, calcium intake, calcium supplements, vitamin D intake, UA levels, alcohol intake, caffeine intake	9
									BMD change	Lumbar spine,			
										Hip			
Tao Zhou^2^	2021	UK	Female & Male	224,630	46.0	56.8 ± 8.0	14.2 ± 6.1	Touchscreen dietary questionnaire	BMD	Heel	Ultrasound	Age, sex, assessment center, BMI, deprivation status, physical activity, smoking status, alcohol intake, dietary calcium intake, dietary vitamin D intake, and total energy intake	9
Taehoon Lee(A^2^	2019	South Korea	Female	319	0.0	> 65	5.31 ± 0.19	24-hr dietary recall data	BMD	Femoral neck,	DXA	Age, BMI, serum vitamin D level, cigarette smoking, physical activity, alcohol use, daily consumption of carbohydrate, protein, fat, calcium, phosphate, iron, thiamine, riboflavin, niacin, and vitamin C, HRT	7
										Lumbar spine,			
										Total femur			
Taehoon Lee(B^2^	2019	South Korea	Male	244	100.0	> 65	9.87 ± 2.33	24-hr dietary recall data	BMD	Femoral neck,	DXA	Age, BMI, serum vitamin D level, cigarette smoking, physical activity, alcohol use, daily consumption of carbohydrate, protein, fat, calcium, phosphate, iron, thiamine, riboflavin, niacin, and vitamin C	7
										Lumbar spine,			
										Total femur			
Zhaoli Dai(A^3^	2018	USA	Female	1,065	0.00	57.3 ± 9.0	19.50 ± 8.10	FFQ	%Δ BMD^1^	Femoral neck,	DXA	Exam period, total energy intake, age, BMI, height, current cigarette smoking, physical activity, modified DGAI 2010 excluding fiber component, calcium supplement intake, vitamin D supplement intake, caffeine intake, dietary calcium, dietary vitamin D, menopausal status, current estrogen use	8
										Lumbar spine,			
										Trochanter			
Zhaoli Dai(B^3^	2018	USA	Male	792	100.0	58.1 ± 8.9	19.70 ± 7.90	FFQ	%Δ BMD	Femoral neck,	DXA	Exam period, total energy intake, age, BMI, height, current cigarette smoking, physical activity, modified DGAI 2010 excluding fiber component, calcium supplement intake, vitamin D supplement intake, caffeine intake, dietary calcium, dietary vitamin D, menopausal status, current estrogen use	8
										Lumbar spine,			
										Trochanter			
LuWei Li^3^	2023	USA	Female & Male	9,871	54.3	50–80	15.20 ± 9.84	24-hr dietary recall data	T-score	Femoral neck,	DXA	Sex, age, family history, height, weight, BMI, caffeine intake, carbohydrate consumption, blood phosphorus, blood potassium, blood sodium	6

^1^BMD, bone mineral density; DXA, dual-energy X-ray absorptiometry; BMI, body mass index; FFQ, food frequency questionnaire; UA levels, uric acid levels; HRT, hormonal replacement therapy; %ΔBMD, percentage change in BMD

^2^Included in meta-analysis

^3^Included in qualitative review

Of the four studies (all with cross-sectional design) involved in the meta-analysis, two reported effects separately for males and females and were therefore treated as independent data points. With a total of six data points, these four studies involved 223,339 individuals (45.3% males; 56.8 $\:\pm\:$ 8.05 years). Specifically, one study was conducted in the US, one in Mexico, one in South Korea, and one in the UK. For the measurement of BMD, three studies used dual-energy X-ray absorptiometry (DXA), a widely recognized standard method, and one used ultrasound. For the measurement of dietary fiber, two studies used the 24-hour dietary recall, one used FFQ, and one used the touchscreen dietary questionnaire following the FFQ design [40, 41].

Three studies were involved in the qualitative review. The two cohort studies were conducted in the US and Mexico, respectively, involving 3,174 individuals (35.3% males; 52.7 $\:\pm\:$ 12.5 years). One cross-sectional study was conducted in the US, involving 9871 individuals (54.3% males; 50–80 years). For the measurement of BMD changes or T-scores, all studies used DXA. For the measurement of dietary fiber, two studies used FFQ and one used the 24-hour dietary recall.

### Risk of bias assessment

Table 1 summarizes the risk of bias assessment for the included studies, with additional details available in Supplementary Tables S3 and S4. All literature was considered of satisfactory quality according to our predefined classification thresholds (at least “moderate”, 100%). Cohort studies were scored between 8 and 9, with primary sources of risk of bias derived from poorly defined baseline outcomes or insufficient follow-up information. Cross-sectional studies were scored between 6 and 9, with primary sources of risk of bias derived from inadequate reporting of missing data handling or no assessment undertaken for quality assurance purposes.

### Association between dietary fiber intake and bone mineral density

**Fig. 2:**
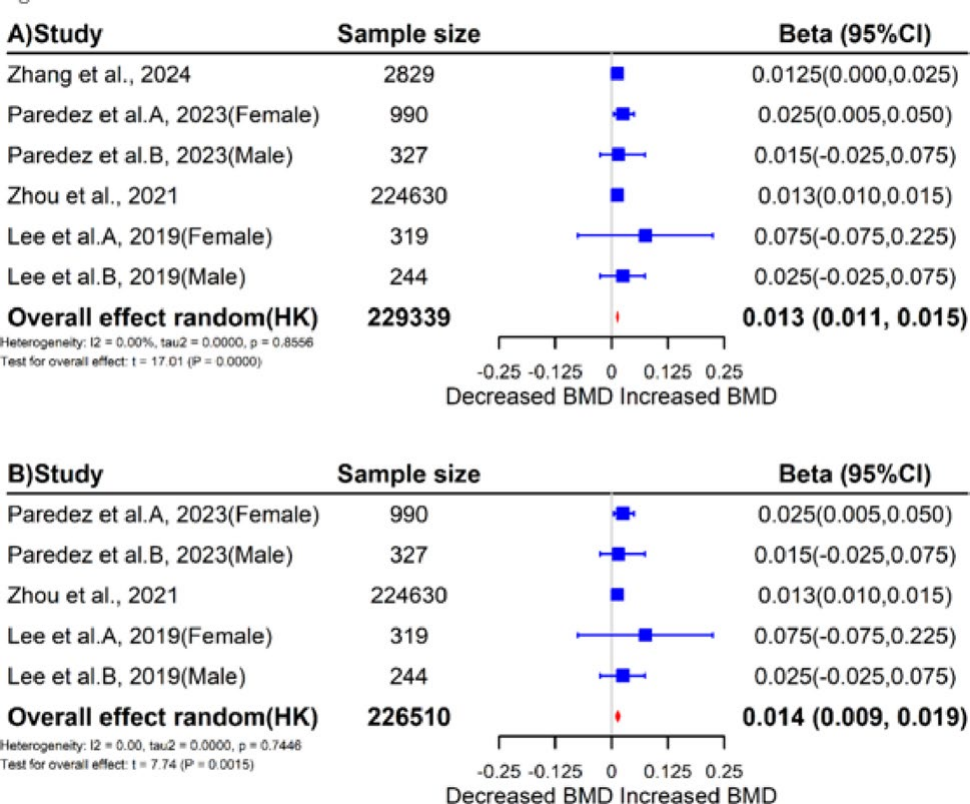
Forest plots of the primary meta-analyses. **A**: The overall meta-analysis of all included studies. **B**: The meta-analysis excluding studies that did not adjust for calcium and vitamin D. Abbreviations – 95%CI, 95% confidence intervals; HK, Hartung Knapp methodmeta-analyses

**Fig. 3:**
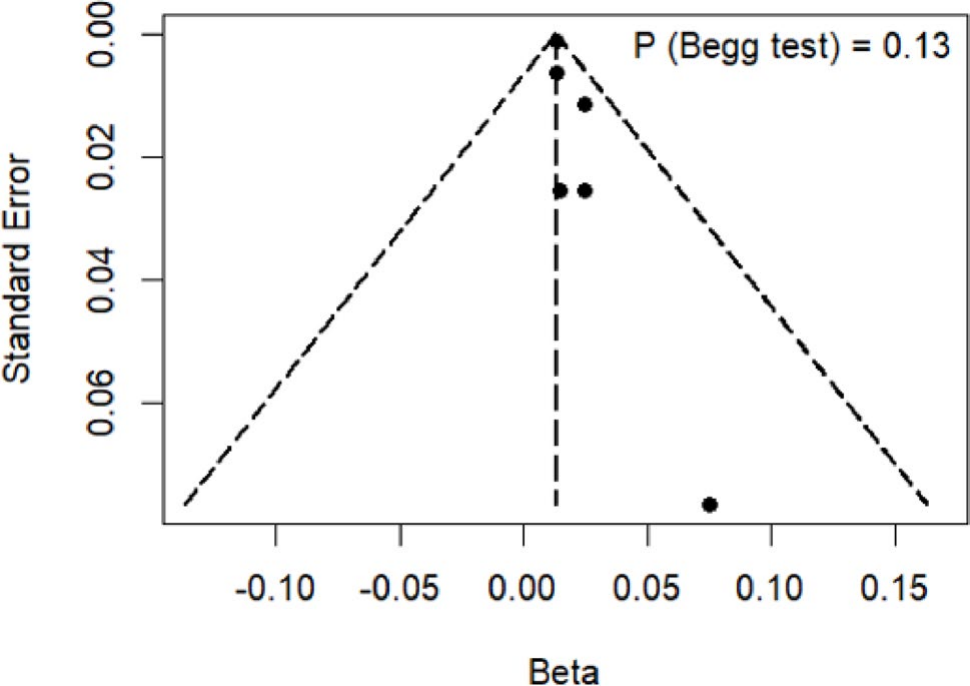
Funnel plots of dietary fiber and BMD

Results of the overall meta-analysis are presented in Fig. 2. A significant positive association was found between dietary fiber intake and BMD level ($\:\beta\:$ = 0.013, 95%CI = 0.011–0.015, $\:P$ < 0.01; $\:{\tau\:}^{2}$ = 0.00; $\:{I}^{2}$ = 0.00%; $\:{P}_{Q-test}$ = 0.86). The association remained significant even in sensitivity analysis excluding studies not adjusting for calcium or vitamin D ($\:\beta\:$ = 0.014, 95%CI = 0.009–0.019, $\:P$ < 0.01; $\:{\tau\:}^{2}$ = 0.00; $\:{I}^{2}$ = 0.00%; $\:{P}_{Q-test}$ = 0.74). Leave-one-out sensitivity analysis confirmed the robustness of results (Supplementary Figure S1). Despite the funnel plot exhibited a non-symmetric distribution, Begg’s test indicated a lack of publication bias ($\:P$ = 0.12) (Fig. 3).

### Subgroup analysis

Details of the subgroup analysis are summarized in Table 2 and Supplementary Figs. S2-S5. Despite effect sizes varied by BMD measurement site, sex and dietary assessment method, subgroup analysis confirmed none as a source of heterogeneity in the association between dietary fiber intake and BMD ($\:{P}_{interaction}$ = 0.31, 0.86 and 0.68, respectively). For sites, despite the effect size being the strongest at lumbar spine ($\:\beta\:$ = 0.025, 95%CI = $\:-$0.049–0.099, $\:P$ = 0.36), statistically significance was observed only at femoral neck ($\:\beta\:$ = 0.018, 95%CI = 0.007–0.030, $\:P$ = 0.01). For sex, the protective effect seemed to be more pronounced in males ($\:\beta\:$ = 0.016, 95%CI = 0.014–0.019, $\:P$ < 0.01) compared to females ($\:\beta\:$ = 0.015, 95%CI = $\:-$0.001–0.031, $\:P$ = 0.06). For dietary assessment method, directionally consistent results were observed for 24-hour dietary recall ($\:\beta\:$ = 0.017, 95% CI = $\:-$0.017–0.051, $\:P$ = 0.16) and FFQ ($\:\beta\:$ = 0.014, 95% CI = 0.004–0.024, $\:P$ = 0.03), while statistically significant result was only observed in the latter.

**Table 2:** Subgroup analysis for studies included in the analysis

Subgroup analysis	*N* ^1^	Sample size	Effect size (95%CI^1^)	I^2^ (%)	τ^2^	*P*_interaction_
Sites of outcomes						
Femoral neck	5	4,709	**0.018(0.007**,**0.030)**	0.00	< 0.01	0.31
Lumbar spine	4	1,880	0.025(-0.049,0.099)	0.00	< 0.01	
Hip	2	1,317	0.009(-0.052,0.070)	0.00	0.00	
Sex						
Female	4	125,433	0.015(-0.001,0.031)	0.00	< 0.01	0.86
Male	3	103,906	**0.016(0.014**,**0.019)**	0.00	0.00	
Region						
Europe and America	2	227,459	**0.013(0.012**,**0.014)**	0.00	< 0.01	0.02
Other	4	1,880	**0.024(0.008**,**0.040)**	0.00	< 0.01	
Dietary assessment method						
24-hr dietary recall	3	33,92	0.017(-0.017,0.051)	0.00	< 0.01	0.68
FFQ^2^	3	225,947	**0.014(0.004**,**0.024)**	0.00	< 0.01	

^1^N, number of studies; 95% CI, 95% confidence intervals.

^2^FFQ, food frequency questionnaire.

Region was the only source of heterogeneity ($\:{P}_{interaction}$ = 0.02). Results of the other region ($\:\beta\:$ = 0.024, 95%CI = 0.008–0.040, $\:P$ = 0.02) showed a stronger effect compared to results of the Europe and America region ($\:\beta\:$ = 0.013, 95%CI = 0.012–0.014, $\:P$ < 0.01).

### Qualitative review

The quantitative results of the meta-analysis suggested a beneficial role of dietary fiber intake on BMD. Such a finding was supported by a study of cross-sectional design but with a special focus on a binary T-score (<$\:\:-$1, indicating BMD abnormality) as the outcome. This study involved 9,871 individuals and reported a modest positive association between dietary fiber intake and the T-score of femoral neck ($\:OR$ = 0.986, 95%CI = 0.980–0.992, $\:P<$0.01).

Furthermore, the Framingham Offspring cohort followed 1,857 individuals (male-to-female ratio 1:1.2) for 12 years, measuring BMD at three sites (femoral neck, lumbar spine, and trochanter) and estimating the effect of dietary fiber intake on percentage BMD change (%ΔBMD) separately by sex. Among males, a significant protective effect was found at the femoral neck ($\:\beta\:$ = 0.06, 95%CI = 0.021–0.099, $\:P$ = 0.003).

In contrast, another study of very similar design – the Health Worker cohort-based study followed 1317 individuals (male-to-female ratio 1:3) for 6 years and measured BMD at three sites (femoral neck, lumbar spine, and hip) – found a modest negative association between dietary fiber intake and BMD change at the femoral neck in males ($\:\beta\:$ = $\:-$0.0007, 95%CI = $\:-$0.001~$\:-$0.00008, $\:P\:<$0.05). For both cohort studies, no significant effect was found at any other site in males nor in females.

## Discussion

To the best of our knowledge, this is the first systematic review and meta-analysis that quantifies the effect of dietary fiber intake on bone health as reflected by BMD. The final meta-analysis included 229,339 participants and demonstrated a higher level of dietary fiber intake to associate with an increased BMD. A possible tendency toward a stronger protective association was observed in males and populations outside Europe and America.

Our overall findings indicate that higher dietary fiber intake is associated with higher BMD. Importantly, we confirmed the results not to be confounded by calcium and vitamin D consumption, as the sensitivity analysis excluding studies lacking relevant adjustments did not alter the results substantially. These findings provide a robust evidence base to inform clinical decision-making and policy development. Moreover, the protective role of dietary fiber is further supported by additional epidemiological evidence, even when the outcomes extended to %ΔBMD and T-score. Evidence synthesized from longitudinal studies suggests that the protective effect is likely to be maintained over a longer period.

Our results indicate that the protective effect of dietary fiber on BMD is more pronounced in specific measurement site and sex. Despite a consistent positive relationship between dietary fiber intake and BMD being observed across all measurement sites, statistical significance was only reached at the femoral neck. This may be due to the relatively limited sample sizes available for lumbar spine or hip ($\:{N}_{lumbar-spine}$ = 1,880; $\:{N}_{hip}$ = 1,317) compared to femoral neck ($\:{N}_{femoral-neck}$ = 4,709), which likely affected the statistical power. The association between dietary fiber intake and BMD was stronger in males than in females, possibly because menopausal estrogen decline accelerates bone loss and weakens bone structure, thereby diminishing the benefits of dietary fiber on BMD [42]. The positive association was statistically significant only within the FFQ subgroup, both a larger sample size ($\:{N}_{FFQ}$ = 225,947; $\:{N}_{24h-recall}$ = 3,392) and a long-term adherence may have contributed to this difference. In addition, the protective effect of dietary fiber on BMD also varied by regions. This may be due to the pronounced regional differences in genetic backgrounds that influence BMD [37]. Moreover, the typical western dietary pattern – high in processed foods, fats, and sweets – can negatively impact BMD by altering the gut microbiome [9]. Similarly, the protective effects of vitamin D3 and vitamin K on BMD are weaker or even non-significant in Caucasian populations compared to other populations [43, 44], suggesting that genetics and dietary patterns may modify nutrient efficacy.

Although the underlying mechanisms remain unclear, dietary fiber – a well-established prebiotic – likely exerts its effects by modulating the gut microbiota. Research has shown that consuming prebiotic fibers alters the composition of gut microbial community, increasing the abundance of specific bacteria such as Lachnospiraceae and Bacteroides [10, 45]. These bacteria ferment dietary fiber to produce short-chain fatty acids (SCFAs), which acidify the intestinal lumen to enhance calcium solubility but may also facilitate calcium absorption by stimulating transcellular transport processes and promoting intestinal morphological adaptations [46, 47]. Additionally, probiotics enhanced by dietary fiber may benefit bone health by supporting maintain a stable bone microenvironment. The underlying mechanisms include synthesizing vitamin K_2_ to promote osteocalcin carboxylation, producing SCFAs to inhibit the NF-κB pathway and reduce osteoclastogenesis, and suppressing the release of bone-resorptive factors (e.g., IL-1β) from macrophages [15, 16]. It is important to note that these processes take time to manifest; therefore, protective effects mediated via the gut-bone axis require prolonged accumulation before becoming observable [17, 18]. This likely explains why the protective effects of dietary fiber on BMD were only identified in the cohort study [25] with an extended follow-up period.

Our findings have significant public health implications. Increasing dietary fiber intake from daily diets benefit BMD, particularly among males and populations outside Europe and America. It is well-recognized that whole-food-derived dietary fiber offers greater benefits in reducing chronic disease risk than that derived from foods fractionated ingredients [48]. Our research thus provides a safe and accessible prevention strategy for populations at high risk of osteoporosis, while expanding the potential health applications of dietary fiber. The results of this meta-analysis offer robust evidence to inform public health policy, health education, and personalized nutritional interventions.

Several limitations should be noted. First, the biological effects of dietary fiber on BMD may vary slightly depending on its food source [22, 25]. Although two studies have examined the impact of dietary fiber from different food sources, their inconsistent outcomes prevented a unified quantitative analysis. Therefore, total dietary fiber intake was chosen as the primary focus of analysis to guarantee sufficient statistical power and to minimize bias. Second, since all studies included in this meta-analysis employed a cross-sectional design, our findings imply an association rather than causation. Third, crucial confounders related to diet and BMD – such as sun exposure and previous fractures – were largely unassessed due to insufficient reporting in most studies. To mitigate this limitation, we incorporated results from the most fully adjusted models available in each study. Fourth, Zhou’s study differed significantly from the other studies included in this meta-analysis in terms of sample size, dietary assessment and BMD measurement method. We attempted to minimize the influence induced by such differences through performing a leave-one-out sensitivity analysis. Results of the sensitivity analysis confirmed the robustness of our main findings. Fifth, although the region-stratified analysis showed variation in the magnitude of associations across subgroups, these findings should be interpreted with caution due to the limited number of studies included in each subgroup.

## Conclusion

This systematic review and meta-analysis of population-based observational studies indicates that higher dietary fiber intake significantly promotes BMD in adults, particularly among males and populations outside Europe and America. Our study not only bridges the gap in quantitative evidence regarding the dietary fiber-BMD relationship but also suggests a potentially stronger positive association between dietary fiber and bone health in specific populations, providing a direction for future targeted interventions. Further large-scale prospective cohort studies are needed to validate these findings.

## Supplementary Information

Below is the link to the electronic supplementary material.

Supplementary Material 1

## Data Availability

The datasets used during the current study are available from the corresponding author on reasonable request.

## References

[1] Yong EL, Logan S (2021) Menopausal osteoporosis: screening, prevention and treatment. Singap Med J 62(4):159–166 10.11622/smedj.2021036 PMC8801823 33948669

[2] Rachner TD, Khosla S, Hofbauer LC (2011) Osteoporosis: now and the future. Lancet 377(9773):1276–1287 21450337 10.1016/S0140-6736(10)62349-5 PMC3555696

[3] Minkin MJ, Menopause (2019) Hormones, Lifestyle, and optimizing aging. Obstet Gynecol Clin N Am 46(3):501–514 10.1016/j.ogc.2019.04.008 31378291

[4] Clynes MA, Harvey NC, Curtis EM, Fuggle NR, Dennison EM, Cooper C (2020) The epidemiology of osteoporosis. Br Med Bull 133(1):105–117 32282039 10.1093/bmb/ldaa005 PMC7115830

[5] Fabiani R, Naldini G, Chiavarini M (2019) Dietary patterns in relation to low bone mineral density and fracture risk: A systematic review and Meta-Analysis. Adv Nutr 10(2):219–236 30657847 10.1093/advances/nmy073 PMC6416046

[6] Levis S, Lagari VS (2012) The role of diet in osteoporosis prevention and management. Curr Osteoporos Rep 10(4):296–302 23001895 10.1007/s11914-012-0119-y

[7] Tucker KL, Hannan MT, Chen H, Cupples LA, Wilson PW, Kiel DP (1999) Potassium, magnesium, and fruit and vegetable intakes are associated with greater bone mineral density in elderly men and women2. Am J Clin Nutr 69(4):727–736 10197575 10.1093/ajcn/69.4.727

[8] Tucker KL, Chen H, Hannan MT, Cupples LA, Wilson PW, Felson D et al (2002) Bone mineral density and dietary patterns in older adults: the Framingham osteoporosis Study1,2,3. Am J Clin Nutr 76(1):245–252 12081842 10.1093/ajcn/76.1.245

[9] Melaku YA, Gill TK, Adams R, Shi Z (2016) Association between dietary patterns and low bone mineral density among adults aged 50 years and above: findings from the North West Adelaide health study (NWAHS). Br J Nutr 116(8):1437–1446 27669817 10.1017/S0007114516003366

[10] Whisner CM, Martin BR, Nakatsu CH, McCabe GP, McCabe LD, Peacock M et al (2014) Soluble maize fibre affects short-term calcium absorption in adolescent boys and girls: a randomised controlled trial using dual stable isotopic tracers. Br J Nutr 112(3):446–456 24848974 10.1017/S0007114514000981

[11] Abrams SA, Griffin IJ, Hawthorne KM, Liang L, Gunn SK, Darlington G et al (2005) A combination of prebiotic short- and long-chain inulin-type Fructans enhances calcium absorption and bone mineralization in young adolescents2, 3. Am J Clin Nutr 82(2):471–476 16087995 10.1093/ajcn.82.2.471

[12] Jakeman SA, Henry CN, Martin BR, McCabe GP, McCabe LD, Jackson GS et al (2016) Soluble corn fiber increases bone calcium retention in postmenopausal women in a dose-dependent manner: a randomized crossover trial1. Am J Clin Nutr 104(3):837–843 27465372 10.3945/ajcn.116.132761

[13] Chen Z, Stini WA, Marshall JR, Martínez ME, Guillén-Rodríguez JM, Roe D et al (2004) Wheat Bran fiber supplementation and bone loss among older people. Nutrition 20(9):747–751 15325680 10.1016/j.nut.2004.05.015

[14] Slevin MM, Allsopp PJ, Magee PJ, Bonham MP, Naughton VR, Strain JJ et al (2014) Supplementation with calcium and short-chain fructo-oligosaccharides affects markers of bone turnover but not bone mineral density in postmenopausal women. J Nutr 144(3):297–304 24453130 10.3945/jn.113.188144

[15] McCabe L, Britton RA, Parameswaran N (2015) Prebiotic and probiotic regulation of bone health: role of the intestine and its Microbiome. Curr Osteoporos Rep 13(6):363–371 26419466 10.1007/s11914-015-0292-x PMC4623939

[16] Feng B, Lu J, Han Y, Han Y, Qiu X, Zeng Z (2024) The role of short-chain fatty acids in the regulation of osteoporosis: new perspectives from gut microbiota to bone health: A review. Med (Baltim) 103(34):e39471 10.1097/MD.0000000000039471 PMC11346881 39183408

[17] Lambert MNT, Thybo CB, Lykkeboe S, Rasmussen LM, Frette X, Christensen LP et al (2017) Combined bioavailable isoflavones and probiotics improve bone status and Estrogen metabolism in postmenopausal osteopenic women: a randomized controlled trial. Am J Clin Nutr 106(3):909–920 28768651 10.3945/ajcn.117.153353

[18] Jafarnejad S, Djafarian K, Fazeli MR, Yekaninejad MS, Rostamian A, Keshavarz SA (2017) Effects of a multispecies probiotic supplement on bone health in osteopenic postmenopausal women: A Randomized, Double-blind, controlled trial. J Am Coll Nutr 36(7):497–506 28628374 10.1080/07315724.2017.1318724

[19] Lee T, Suh HS (2019) Associations between dietary fiber intake and bone mineral density in adult Korean population: analysis of National health and nutrition examination survey in 2011. J Bone Metab 26(3):151–160 31555612 10.11005/jbm.2019.26.3.151 PMC6746664

[20] Sasaki S, Yanagibori R (2001) Association between current nutrient intakes and bone mineral density at calcaneus in pre- and postmenopausal Japanese women. J Nutr Sci Vitaminol (Tokyo) 47(4):289–294 11767209 10.3177/jnsv.47.289

[21] Zhang L, Zhao L, Xiao X, Zhang X, He L, Zhang Q (2024) Association of dietary carbohydrate and fiber ratio with postmenopausal bone mineral density and prevalence of osteoporosis: A cross-sectional study. PLoS ONE 19(2):e0297332 38354209 10.1371/journal.pone.0297332 PMC10866481

[22] Zhou T, Wang M, Ma H, Li X, Heianza Y, Qi L (2021) Dietary Fiber, genetic variations of gut Microbiota-derived Short-chain fatty Acids, and bone health in UK biobank. J Clin Endocrinol Metab 106(1):201–210 33051670 10.1210/clinem/dgaa740 PMC8186524

[23] Li L, Cheng S, Xu G (2023) Application of neural network and nomogram for the prediction of risk factors for bone mineral density abnormalities: A cross-sectional NHANES-based survey. Heliyon 9(10):e20677 37829807 10.1016/j.heliyon.2023.e20677 PMC10565773

[24] Rivera-Paredez B, León-Reyes G, Rangel-Marín D, Salmerón J, Velázquez-Cruz R (2023) Associations between macronutrients intake and bone mineral density: A longitudinal analysis of the health workers cohort study participants. J Nutr Health Aging 27(12):1196–1205 38151870 10.1007/s12603-023-2038-2 PMC12880493

[25] Dai Z, Zhang Y, Lu N, Felson DT, Kiel DP, Sahni S (2018) Association between dietary fiber intake and bone loss in the Framingham offspring study. J Bone Min Res 33(2):241–249 10.1002/jbmr.3308 PMC5990003 29024045

[26] Stang A (2010) Critical evaluation of the Newcastle-Ottawa scale for the assessment of the quality of nonrandomized studies in meta-analyses. Eur J Epidemiol 25(9):603–605 20652370 10.1007/s10654-010-9491-z

[27] Ma LL, Wang YY, Yang ZH, Huang D, Weng H, Zeng XT (2020) Methodological quality (risk of bias) assessment tools for primary and secondary medical studies: what are they and which is better? Mil Med Res 7(1):7 32111253 10.1186/s40779-020-00238-8 PMC7049186

[28] Hou J, Deng Q, Sha L, Zhu J, Xiang R, Zhao X et al (2025) Physical activity and risk of depression in adolescents: A systematic review and meta-analysis of prospective observational studies. J Affect Disord 371:279–288 39581382 10.1016/j.jad.2024.11.065

[29] Wang W, Wu C, Bai D, Chen H, Cai M, Gao J et al (2022) A meta-analysis of nursing students’ knowledge and attitudes about end-of-life care. Nurse Educ Today 119:105570 36182790 10.1016/j.nedt.2022.105570

[30] Carbohydrate intake for adults and children: WHO guideline 37490573

[31] Reid IR, Bolland MJ, Grey A (2014) Effects of vitamin D supplements on bone mineral density: a systematic review and meta-analysis. Lancet 383(9912):146–155 24119980 10.1016/S0140-6736(13)61647-5

[32] Higgins JPT, Thompson SG, Deeks JJ, Altman DG (2003) Measuring inconsistency in meta-analyses. BMJ 327(7414):557–560 12958120 10.1136/bmj.327.7414.557 PMC192859

[33] Dogo SH, The effect of some estimators of between-study variance (2017) on random-effects meta-analysis. Sci World J 12(2):14–18

[34] Röver C, Knapp G, Friede T (2015) Hartung-Knapp-Sidik-Jonkman approach and its modification for random-effects meta-analysis with few studies. BMC Med Res Methodol 15:99 26573817 10.1186/s12874-015-0091-1 PMC4647507

[35] Karasik D, Ferrari SL (2008) Contribution of gender-specific genetic factors to osteoporosis risk. Ann Hum Genet 72(Pt 5):696–714 18485052 10.1111/j.1469-1809.2008.00447.x

[36] Gaskins AJ, Mumford SL, Zhang C, Wactawski-Wende J, Hovey KM, Whitcomb BW et al (2009) Effect of daily fiber intake on reproductive function: the biocycle study. Am J Clin Nutr 90(4):1061–1069 19692496 10.3945/ajcn.2009.27990 PMC2744625

[37] Massart F, Brandi ML (2007) Bone mass pharmacogenetics and ethnic health implications. Clin Cases Min Bone Metab 4(2):131–138 PMC2781242 22461213

[38] Chen X, Fu Y, Zhu Z (2025) Association between dietary protein intake and bone mineral density based on NHANES 2011–2018. Sci Rep 15(1):8638 40082692 10.1038/s41598-025-93642-w PMC11906839

[39] Zhang R, Huang Q, Su G, Wei M, Cui Y, Zhou H et al (2023) Association between multiple vitamins and bone mineral density: a cross-sectional and population-based study in the NHANES from 2005 to 2006. BMC Musculoskelet Disord 24(1):113 36765290 10.1186/s12891-023-06202-6 PMC9912521

[40] Bradbury KE, Young HJ, Guo W, Key TJ (2018) Dietary assessment in UK biobank: an evaluation of the performance of the touchscreen dietary questionnaire. J Nutr Sci 7:e6 29430297 10.1017/jns.2017.66 PMC5799609

[41] Health research data for the world. UK Biobank

[42] Minkin MJ, Menopause (2019) Hormones, Lifestyle, and optimizing aging. Obstet Gynecol Clin North Am 46(3):501–514 31378291 10.1016/j.ogc.2019.04.008

[43] Kazemian E, Pourali A, Sedaghat F, Karimi M, Basirat V, Sajadi Hezaveh Z et al (2023) Effect of supplemental vitamin D3 on bone mineral density: a systematic review and meta-analysis. Nutr Rev 81(5):511–530 36308775 10.1093/nutrit/nuac068

[44] Fang Y, Hu C, Tao X, Wan Y, Tao F (2012) Effect of vitamin K on bone mineral density: a meta-analysis of randomized controlled trials. J Bone Min Metab 30(1):60–68 10.1007/s00774-011-0287-3 21674202

[45] Weaver CM, Martin BR, Nakatsu CH, Armstrong AP, Clavijo A, McCabe LD et al (2011) Galactooligosaccharides improve mineral absorption and bone properties in growing rats through gut fermentation. J Agric Food Chem 59(12):6501–6510 21553845 10.1021/jf2009777

[46] Cashman K (2003) Prebiotics and calcium bioavailability. Curr Issues Intest Microbiol 4(1):21–32 12691259

[47] Lin X, Yu Z, Liu Y, Li C, Hu H, Hu JC et al (2025) Gut–X axis. iMeta 4(1):e270 40027477 10.1002/imt2.270 PMC11865426

[48] Thompson HJ (2021) The dietary guidelines for Americans (2020–2025): Pulses, dietary Fiber, and chronic disease Risk—A call for clarity and action. Nutrients 13(11):4034 34836289 10.3390/nu13114034 PMC8621412

