# Supplementary material for: Association between dietary fiber intake and bone mineral density: a systematic review and meta-analysis of observational studies
Source: Eur J Nutr. 2026 Feb 25;65(2):70. doi: 10.1007/s00394-025-03866-9 (PMC12935702; doi:10.1007/s00394-025-03866-9)
Supplement: Supplementary file 1 — Supplementary Material 1 [file 394_2025_3866_MOESM1_ESM.docx]

| **Table S1. Search strategy** | |
| --- | --- |
| **Ovid** | |
| 1 | exp dietary fiber/ |
| 2 | "diet*".af. |
| 3 | (fiber* or fibre*).ab,ti. |
| 4 | 2 and 3 |
| 5 | 1 or 4 |
| 6 | (bone* and (mineral* or mass* or densit*)).ab,ti. |
| 7 | exp bone mineral density/ |
| 8 | 6 or 7 |
| 9 | (population* or subject* or individual* or epidemic*).af. |
| 10 | 5 and 8 and 9 |
| 11 | limit 10 to english language [Limit not valid in Journals@Ovid,Your Journals@Ovid,ACP Journal Club,CDSR,CCA,CLCMR,DARE; records were retained] |
| 12 | limit 11 to yr="2000 - 2025" [Limit not valid in DARE; records were retained] |
| **PubMed** | |
| 1 | "diet*"[All Fields] |
| 2 | "fiber*"[Title/Abstract] OR "fibre*"[Title/Abstract] |
| 3 | "diet*"[All Fields] AND ("fiber*"[Title/Abstract] OR "fibre*"[Title/Abstract]) |
| 4 | "bone*"[Title/Abstract] |
| 5 | "mineral*"[All Fields] OR "mass*"[All Fields] OR "densit*"[All Fields] |
| 6 | "bone*"[Title/Abstract] AND ("mineral*"[All Fields] OR "mass*"[All Fields] OR "densit*"[All Fields]) |
| 7 | "population*"[All Fields] OR "subject*"[All Fields] OR "individual*"[All Fields] OR "epidemic*"[All Fields] |
| 8 | #3 AND #6 AND #7 |
| 9 | #8 AND (2000:3000/12/12[pdat]) |
| **Web of Science** | |
| 1 | TS=(diet*) |
| 2 | TI=(fiber* OR fibre*) OR AB=(fiber* OR fibre*) |
| 3 | #1 AND #2 |
| 4 | TI=(bone*) OR AB=(bone*) |
| 5 | TS=(mineral* OR mass* OR densit*) |
| 6 | #4 AND #5 |
| 7 | TS=(population* OR subject* OR individual* OR epidemic*) |
| 8 | #3 AND #6 AND #7 |
| 9 | TS=(diet*) AND (TI=(fiber* OR fibre*) OR AB=(fiber* OR fibre*)) AND (TI=(bone*) OR AB=(bone*)) AND TS=(mineral* OR mass* OR densit*) AND TS=(population* OR subject* OR individual* OR epidemic*) and Preprint Citation Index (Exclude – Database) and 2024 or 2023 or 2022 or 2021 or 2020 or 2019 or 2018 or 2017 or 2016 or 2015 or 2014 or 2013 or 2012 or 2011 or 2010 or 2009 or 2008 or 2007 or 2006 or 2005 or 2004 or 2003 or 2002 or 2001 or 2000 (Publication Years) |
| **Sciencedirect** | |
| 1 | (Dietary) AND (population OR subject OR individual OR epidemic) Year: 2000-2025 Title, abstract, keywords: (fiber OR fibre) AND ((Bone) AND(density OR Mineral OR mass)) Article type: Review articles/Research articles |

| **Table S2. List of studies included and excluded via full-text assessment.** |
| --- |
| 1. Ineligible publication type^1-24^ |
| 1. Ineligible exposure^25-38^ |
| 1. Ineligible outcome^39-47^ |
| 1. Ineligible subjects^48-55^ |
| 1. No available data^56-58^ |
| 1. duplicable sample^59^ |

| **Table S3. The quality assessment of the included cohort studies.** | | | | | | | | | | |
| --- | --- | --- | --- | --- | --- | --- | --- | --- | --- | --- |
| Study | Selection | | | | Comparability† | Outcome | | | Scores | Quality |
|  | representativeness of the exposed cohort | selection of the non-exposed cohort | ascertainment of exposure | demonstration that outcome of interest was no present at start of study | control  important factors | assessment of outcome | follow-up long enough for outcomes to occur‡ | adequacy of follow up of cohort § |  |  |
| Rivera-Paredez et al., 2023^60^ | * | * | * | * | ** | * | * | * | 9 | High |
| Dai et al., 2018^61^ | * | * | * | * | ** | * | * | - | 8 | High |
| *A study could be awarded a maximum of one star for each item except for the item Control for important factor or additional factor.  † A maximum of 2 stars could be awarded for this item. Studies that controlled for calcium and vitamin D received one star, whereas studies that controlled for other important confounders such as age, Socio-demography, total energy intake et al received an additional star.  ‡ A cohort study with a follow-up time >5 years was assigned one star.  § A cohort study with a follow-up rate >85% or description of these lost was assigned one star. | | | | | | | | | | |

| **Table S4. The quality assessment of the included cross-sectional studies.** | | | | | |
| --- | --- | --- | --- | --- | --- |
| Criteria | Zhang et al., 2024^62^ | Rivera-Paredez et al., 2023^60^ | Li et al., 2023^63^ | Zhou et al., 2021^64^ | Lee et al., 2019^65^ |
| 1. Define the source of information (survey, record review). | Y | Y | Y | Y | Y |
| 2. List inclusion and exclusion criteria for exposed and unexposed subjects (cases and controls) or refer to previous publications. | Y | Y | Y | Y | Y |
| 3. Indicate time period used for identifying patients. | Y | Y | Y | Y | Y |
| 4. Indicate whether or not subjects were consecutive if not population-based. | Y | Y | Y | Y | Y |
| 5. Indicate if evaluators of subjective components of study were masked to other aspects of the status of the participants. | U | U | U | U | U |
| 6. Describe any assessments undertaken for quality assurance purposes (e.g., test/retest of primary outcome measurements). | Y | Y | N | Y | N |
| 7. Explain any patient exclusions from analysis. | Y | Y | N | Y | Y |
| 8. Describe how confounding was assessed and/or controlled. | Y | Y | Y | Y | Y |
| 9. If applicable, explain how missing data were handled in the analysis. | Y | N | Y | Y | N |
| 10. Summarize patient response rates and completeness of data collection. | Y | Y | N | Y | Y |
| 11. Clarify what follow-up, if any, was expected and the percentage of patients for which incomplete data or follow-up was obtained. | U | Y | U | U | U |
| Total score | 9 | 9 | 6 | 9 | 7 |
| Quality | H | H | M | H | M |
| Note: Y, yes; N, no; U, unclear; H, high quality; M, medium quality. | | | | | |


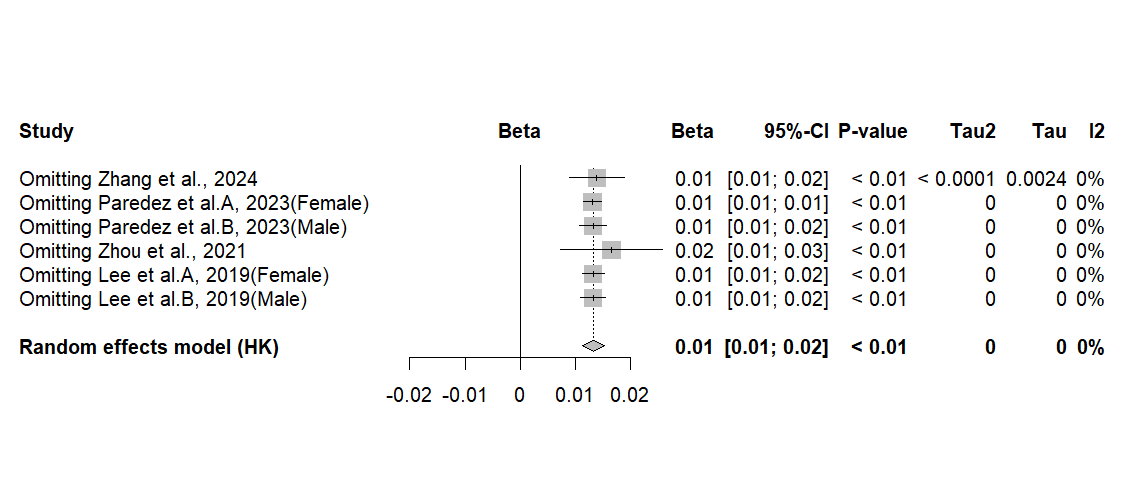


Figure S1. Sensitivity analysis for overall effect.

95%CI, 95% confidence intervals; HK, Hartung Knapp method.


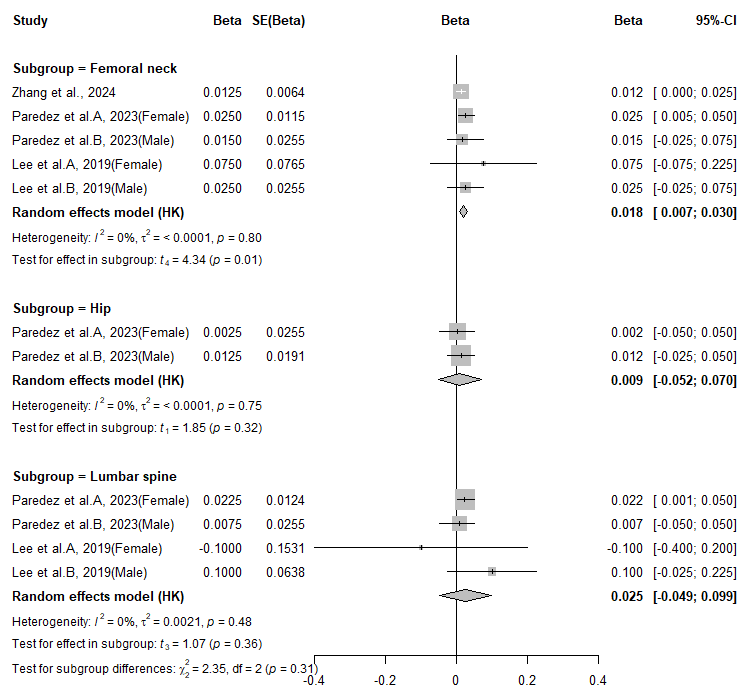


Figure S2. Subgroup analysis of association between dietary fiber intake and bone mineral density by site of measurement.

95%CI, 95% confidence intervals; HK, Hartung-Knap-Sidik-Jonkman method.


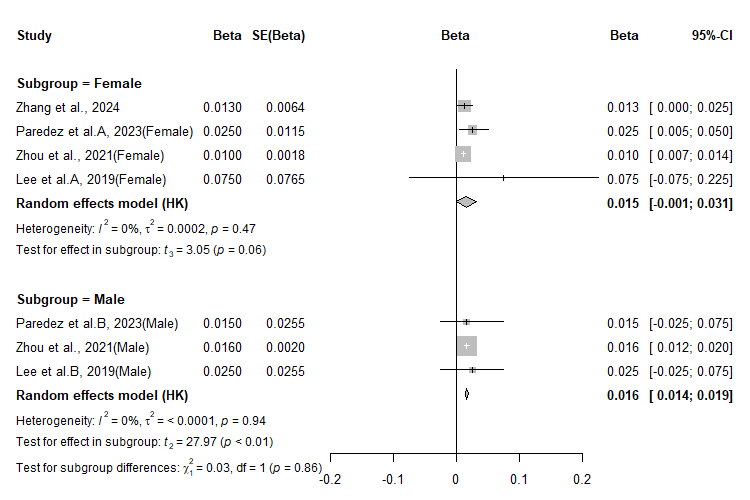


Figure S3. Subgroup analysis of association between dietary fiber intake and bone mineral density by sex.

95%CI, 95% confidence intervals; HK, Hartung-Knap-Sidik-Jonkman method.


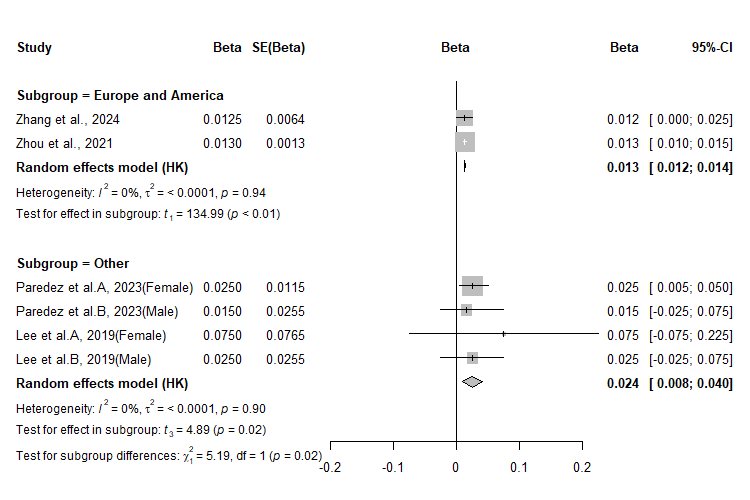


Figure S4. Subgroup analysis of association between dietary fiber intake and bone mineral density by region.

95%CI, 95% confidence intervals; HK, Hartung-Knap-Sidik-Jonkman meth.


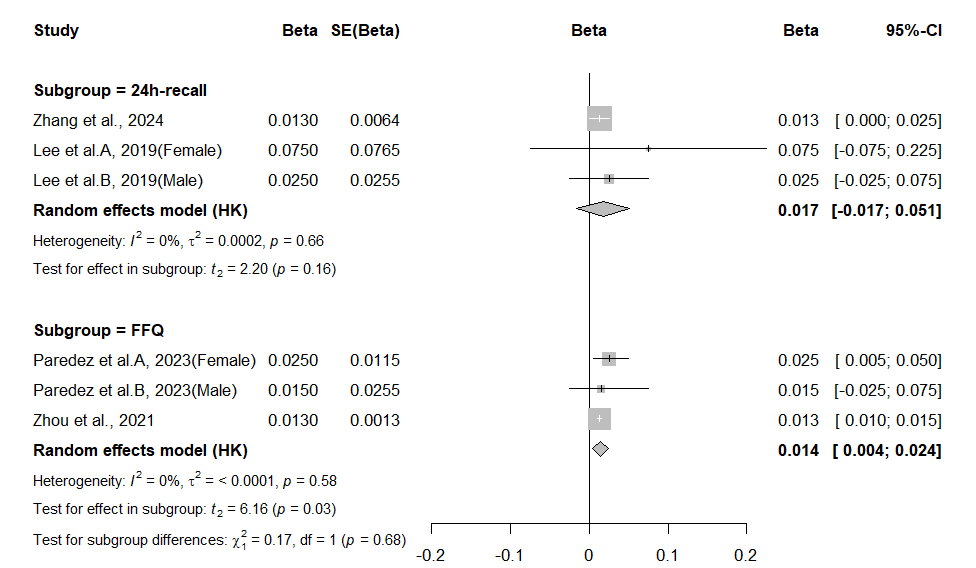
Figure S5. Subgroup analysis of association between dietary fiber intake and bone mineral density by dietary assessment method.

95%CI, 95% confidence intervals; HK, Hartung-Knap-Sidik-Jonkman meth; FFQ, food frequency questionnaire.

**Reference**

1. Biver E, Herrou J, Larid G, et al. Dietary recommendations in the prevention and treatment of osteoporosis. *Joint Bone Spine*. 01 May 2023;90(3) (no pagination)105521.

2. Dai Z, Hirani V, Sahni S, et al. Association of dietary fiber and risk of hip fracture in men from the Framingham Osteoporosis Study and the Concord Health and Ageing in Men Project. *Nutrition and Health*. 2022;28(2):229-238.

3. Nithya VS, Athul TP, Deogade M, Nesari TM. Role of foxtail millet (Setaria italica (L.) P. Beauvois) in senile bone degenerative diseases: A conceptual review. *Journal of Drug Research in Ayurvedic Sciences*. 2023;8(Suppl 1) Supplement(1):S76-S81.

4. Tucker KL. Dietary intake and bone status with aging. *Curr Pharm Des*. 2003;9(32):2687-704.

5. Tabatabai LS, Sellmeyer DE. Nutritional Supplements and Skeletal Health. Review. *Current Osteoporosis Reports*. 01 Feb 2021;19(1):23-33.

6. Schaafsma G, Slavin JL. Significance of Inulin Fructans in the Human Diet. *Compr Rev Food Sci Food Saf*. Jan 2015;14(1):37-47.

7. Caers W. Inulin and oligofructose: their effects on mineral absorption and bone health - a review on recent studies. 2006:247-256.

8. Wallace TC, Marzorati M, Spence L, Weaver CM, Williamson PS. New Frontiers in Fibers: Innovative and Emerging Research on the Gut Microbiome and Bone Health. *J Am Coll Nutr*. Mar-Apr 2017;36(3):218-222.

9. Feng B, Lu J, Han Y, Han Y, Qiu X, Zeng Z. The role of short-chain fatty acids in the regulation of osteoporosis: new perspectives from gut microbiota to bone health: A review. *Medicine (Baltimore)*. Aug 23 2024;103(34):e39471.

10. Hughes RL, Alvarado DA, Swanson KS, Holscher HD. The Prebiotic Potential of Inulin-Type Fructans: A Systematic Review. *Advances in Nutrition*. 2022;13(2):492-529.

11. Neufingerl N, Eilander A. Nutrient intake and status in adults consuming plant-based diets compared to meat-eaters: A systematic review. Review. *Nutrients*. 01 Jan 2022;14(1) (no pagination)29.

12. Hassoon A, Michos ED, Miller ER, III, Crisp Z, Appel LJ. Effects of Different Dietary Interventions on Calcitriol, Parathyroid Hormone, Calcium, and Phosphorus: Results from the DASH Trial. Article. *Nutrients*. Mar 2018;10(3)367.

13. Sheean P, Kabir C, Rao R, Hoskins K, Stolley M. Exploring Diet, Physical Activity, and Quality of Life in Females with Metastatic Breast Cancer: A Pilot Study to Support Future Intervention. *J Acad Nutr Diet*. Oct 2015;115(10):1690-8.

14. Shon J, Seong Y, Choi Y, et al. Meal-Based Intervention on Health Promotion in Middle-Aged Women: A Pilot Study. *Nutrients*. 01 May 2023;15(9) (no pagination)2108.

15. Chen Z, Stini WA, Marshall JR, et al. Wheat bran fiber supplementation and bone loss among older people. *Nutrition*. 2004/09/01/ 2004;20(9):747-751.

16. Whisner CM, Martin BR, Nakatsu CH, et al. Soluble Corn Fiber Increases Calcium Absorption Associated with Shifts in the Gut Microbiome: A Randomized Dose-Response Trial in Free-Living Pubertal Females. Article. *Journal of Nutrition*. Jul 2016;146(7):1298-1306.

17. Jakeman SA, Henry CN, Martin BR, et al. Soluble corn fiber increases bone calcium retention in postmenopausal women in a dose-dependent manner: a randomized crossover trial. Article. *American Journal of Clinical Nutrition*. Sep 2016;104(3):837-843.

18. Hirota T, Hirota K. [Diet for lifestyle-related diseases to maintain bone health]. *Clin Calcium*. May 2011;21(5):730-6.

19. Jang J-H, Yoon J-Y, Cho S-H. Intake of dietary phytoestrogen and indices of antioxidant and bone metabolism of pre- and post-menopausal Korean women. *Nutrition research and practice*. 2007 2007;1(4):305-12.

20. Lorincz C, Manske SL, Zernicke R. Bone health: part 1, nutrition. *Sports Health*. May 2009;1(3):253-60.

21. Miggiano GA, Gagliardi L. [Diet, nutrition and bone health]. *Clin Ter*. Jan-Apr 2005;156(1-2):47-56. Dieta, nutrizione e salute dell'osso.

22. Millward DJ. Interactions between Growth of Muscle and Stature: Mechanisms Involved and Their Nutritional Sensitivity to Dietary Protein: The Protein-Stat Revisited. Review. *Nutrients*. Mar 2021;13(3)729.

23. Reeve J, Abraham R, Walton J, Russell L, Wardley-Smith B, Mitchell A. Increasing mineral density after menopause in individual lumbar vertebrae as a marker for incident degenerative disease: A pilot study for the effects of body composition and diet. *Journal of Rheumatology*. October 2004;31(10):1986-1992.

24. Schraders K, Coad J, Kruger M. Bone Health in Premenopausal Women with Coeliac Disease: An Observational Study. *Nutrients*. 01 Jul 2024;16(14) (no pagination)2178.

25. Bakirhan H, Yildiran H, Cankay TU. Dietary patterns and migraine: are dietary intake and biochemical parameters associated with migraine characteristics? Article. *Nutrition & Food Science*. Feb 28 2023;53(3):630-645.

26. Song D, Kim J, Kang M, et al. Association between the dietary inflammatory index and bone markers in postmenopausal women. *PLoS ONE*. 01 Mar 2022;17(3 March) (no pagination)e0265630.

27. Tay W, Quek R, Lim J, Kaur B, Ponnalagu S, Henry CJ. Plant-based alternative proteins-are they nutritionally more advantageous? Article. *European Journal of Clinical Nutrition*. Nov 2023;77(11):1051-1060.

28. Ilich JZ, Cvijetic S, Baric IC, et al. Nutrition and lifestyle in relation to bone health and body weight in Croatian postmenopausal women. *Int J Food Sci Nutr*. Jun 2009;60(4):319-32.

29. Wernicke C, Apostolopoulou K, Hornemann S, et al. Long-Term effects of a food pattern on cardiovascular risk factors and age-related changes of muscular and cognitive function. *Medicine (United States)*. 25 Sep 2020;99(39):E22381.

30. Batool I, Altemimi AB, Munir S, et al. Exploring flaxseed's potential in enhancing bone health: Unveiling osteo-protective properties. *Journal of Agriculture and Food Research*. 2024/03/01/ 2024;15:101018.

31. Li S, Zeng M. The association between dietary inflammation index and bone mineral density: results from the United States National Health and nutrition examination surveys. *Renal Failure*. 2023;45(1) (no pagination)2209200.

32. Mazidi M, Kengne AP, Vatanparast H. Association of dietary patterns of American adults with bone mineral density and fracture. *Public health nutrition*. 01 Sep 2018;21(13):2417-2423.

33. Melaku YA, Gill TK, Adams R, Shi Z. Association between dietary patterns and low bone mineral density among adults aged 50 years and above: Findings from the North West Adelaide Health Study (NWAHS). *British Journal of Nutrition*. 28 Oct 2016;116(8):1437-1446.

34. Runting H, Qingyue L, Yining Y, Huiyu S, Shu Y, Xixi F. Is bone mineral density in middle-aged and elderly individuals associated with their dietary patterns? A study based on NHANES. *Front Nutr*. 2024;11:1396007.

35. Grygiel-Gorniak B, Przyslawski J, Puszczewicz M, Marcinkowska J. THU0416 Nutritional Risk Factors of Postmenopausal Osteoporosis. *Annals of the Rheumatic Diseases*. 2013/06/01/ 2013;72:A305.

36. Karamati M, Yousefian-Sanni M, Shariati-Bafghi S-E, Rashidkhani B. Major Nutrient Patterns and Bone Mineral Density among Postmenopausal Iranian Women. Article. *Calcified Tissue International*. Jun 2014;94(6):648-658.

37. Melaku YA, Gill TK, Taylor AW, Adams R, Shi Z. Association between nutrient patterns and bone mineral density among ageing adults. *Clinical Nutrition ESPEN*. 01 Dec 2017;22:97-106.

38. de Jonge EAL, Koromani F, Hofman A, et al. Dietary acid load, trabecular bone integrity, and mineral density in an ageing population: the Rotterdam study. *Osteoporosis International*. 01 Aug 2017;28(8):2357-2365.

39. Masse PG, Dosy J, Tranchant CC, Dallaire R. Dietary macro- and micronutrient intakes of nonsupplemented pre- and postmenopausal women with a perspective on menopause-associated diseases. *Journal of Human Nutrition and Dietetics*. April 2004;17(2):121-132.

40. Jimenez-Ortega RF, Aparicio-Bautista DI, Becerra-Cervera A, et al. Association Study between Antioxidant Nutrient Intake and Low Bone Mineral Density with Oxidative Stress-Single Nucleotide Variants: GPX1 (rs1050450 and rs17650792), SOD2 (rs4880) and CAT (rs769217) in Mexican Women. Article. *Antioxidants*. Dec 2023;12(12)2089.

41. Fuglsang-Nielsen R, Rakvaag E, Vestergaard P, Hermansen K, Gregersen S, Starup-Linde J. The Effects of 12-Weeks Whey Protein Supplements on Markers of Bone Turnover in Adults With Abdominal Obesity &ndash; A Post Hoc Analysis. Journal article. *Frontiers in endocrinology*. 2022;13

42. Faraj M, Leanza G, Krug J, et al. High-fiber diet reduces bone formation but does not affect bone microarchitecture in type 2 diabetes individuals. Article. *Jbmr Plus*. Sep 9 2024;8(10)ziae111.

43. Wang Y, Xie D, Li J, et al. Association between dietary selenium intake and the prevalence of osteoporosis: a cross-sectional study. *BMC Musculoskelet Disord*. Dec 4 2019;20(1):585.

44. Gvozdenovic N, Sarac I, Coric A, et al. Impact of Vitamin D Status and Nutrition on the Occurrence of Long Bone Fractures Due to Falls in Elderly Subjects in the Vojvodina Region of Serbia. Article. *Nutrients*. Aug 2024;16(16)2702.

45. Key TJ, Papier K, Tong TYN. Plant-based diets and long-term health: findings from the EPIC-Oxford study. *Proc Nutr Soc*. May 2022;81(2):190-198.

46. Valtueña S, Sette S, Branca F. Influence of Mediterranean diet and Mediterranean lifestyle on calcium and bone metabolism. Article; Proceedings Paper. *International Journal for Vitamin and Nutrition Research*. May 2001;71(3):189-202.

47. Alissa EM, Qadi SG, Alhujaili NA, Alshehri AM, Ferns GA. Effect of diet and lifestyle factors on bone health in postmenopausal women. *Journal of Bone and Mineral Metabolism*. November 2011;29(6):725-735.

48. Dreher ML. Whole Fruits and Fruit Fiber Emerging Health Effects. *Nutrients*. Nov 28 2018;10(12)

49. Lim H, Kim HJ, Hong SJ, Kim S. Nutrient intake and bone mineral density by nutritional status in patients with inflammatory bowel disease. *J Bone Metab*. Aug 2014;21(3):195-203.

50. Miazgowski T, Krzyzanowska-Swiniarska B, Ogonowski J, Noworyta-Zietara M. [Does type 2 diabetes predispose to osteoporotic bone fractures?]. *Endokrynol Pol*. May-Jun 2008;59(3):224-9. Czy cukrzyca typu 2 predysponuje do osteoporotycznych złamań kości?

51. Shin Y, In-Sook K, Woon Y, Kim Y. Relationship Between Nutrient Intake and Bone Mineral Density in 20～30 Year-old Korean Women. research-article. *Preventive Nutrition and Food Science*. 2009 2009;14(3):208-213.

52. Kvammen JA, Thomassen RA, Kjeserud CN, et al. Bone mineral density and vitamin D in paediatric intestinal failure patients receiving home parenteral nutrition. *Clinical Nutrition ESPEN*. 01 Oct 2020;39:234-241.

53. Braun TP, Orwoll B, Zhu X, et al. Regulation of lean mass, bone mass, and exercise tolerance by the central melanocortin system. *PLoS One*. 2012;7(7):e42183.

54. Ephraim E, Jewell DE. Betaine and Soluble Fiber Improve Body Composition and Plasma Metabolites in Cats with Chronic Kidney Disease. Journal article. *Frontiers in bioscience (Elite edition)*. 2023;Vol.15(2):8p.

55. Kindler J, Khoury P, Urbina E, Zemel B. Dietary Fiber and Bone Density in Youth with Type 2 Diabetes. *Current Developments in Nutrition*. 2020/06/01/ 2020;4:nzaa063_047.

56. Farrell VA, Harris M, Lohman TG, et al. Comparison between Dietary Assessment Methods for Determining Associations between Nutrient Intakes and Bone Mineral Density in Postmenopausal Women. *Journal of the American Dietetic Association*. 2009/05/01/ 2009;109(5):899-904.

57. Sasaki S, Yanagibori R. Association between current nutrient intakes and bone mineral density at calcaneus in pre- and postmenopausal Japanese women. Article. *Journal of Nutritional Science and Vitaminology*. Aug 2001;47(4):289-294.

58. Atlantis E, Martin SA, Haren MT, Taylor AW, Wittert GA, Florey Adelaide Male Aging S. Lifestyle factors associated with age-related differences in body composition: the Florey Adelaide Male Aging Study. Article. *American Journal of Clinical Nutrition*. Jul 2008;88(1):95-104.

59. Frampton J, Murphy KG, Frost G, Chambers ES. Higher dietary fibre intake is associated with increased skeletal muscle mass and strength in adults aged 40 years and older. *Journal of Cachexia, Sarcopenia and Muscle - Open Access*. 2021;12(6):2134-2144.

60. Rivera-Paredez B, Leon-Reyes G, Rangel-Marin D, Salmeron J, Velazquez-Cruz R. Associations between Macronutrients Intake and Bone Mineral Density: A Longitudinal Analysis of the Health Workers Cohort Study Participants. Journal of Nutrition, Health and Aging. 01 Dec 2023;27(12):1196-1205.

61. Dai Z, Zhang Y, Lu N, Felson DT, Kiel DP, Sahni S. Association Between Dietary Fiber Intake and Bone Loss in the Framingham Offspring Study. J Bone Miner Res. Feb 2018;33(2):241-249.

62. Zhang L, Zhao L, Xiao X, Zhang X, He L, Zhang Q. Association of dietary carbohydrate and fiber ratio with postmenopausal bone mineral density and prevalence of osteoporosis: A cross-sectional study. PLoS ONE. 2024;19(2 February)

63. Li L, Cheng S, Xu G. Application of neural network and nomogram for the prediction of risk factors for bone mineral density abnormalities: A cross-sectional NHANES-based survey. Heliyon. Oct 2023;9(10):e20677.

64. Zhou T, Wang M, Ma H, Li X, Heianza Y, Qi L. Dietary Fiber, Genetic Variations of Gut Microbiota-derived Short-chain Fatty Acids, and Bone Health in UK Biobank. J Clin Endocrinol Metab. Jan 1 2021;106(1):201-210.

65. Lee T, Suh HS. Associations between dietary fiber intake and bone mineral density in adult Korean population: Analysis of national health and nutrition examination survey in 2011. Journal of Bone Metabolism. 01 Aug 2019;26(3):151-160.
